# Predicting the Distribution of Commercially Important Invertebrate Stocks under Future Climate

**DOI:** 10.1371/journal.pone.0046554

**Published:** 2012-12-12

**Authors:** Bayden D. Russell, Sean D. Connell, Camille Mellin, Barry W. Brook, Owen W. Burnell, Damien A. Fordham

**Affiliations:** 1 Southern Seas Ecology Laboratories, School of Earth & Environmental Sciences, University of Adelaide, Adelaide, South Australia, Australia; 2 Australian Institute of Marine Science, Townsville, Queensland, Australia; 3 The Environment Institute and School of Earth & Environmental Sciences, University of Adelaide, Adelaide, South Australia, Australia; University of Western Australia, Australia

## Abstract

The future management of commercially exploited species is challenging because techniques used to predict the future distribution of stocks under climate change are currently inadequate. We projected the future distribution and abundance of two commercially harvested abalone species (blacklip abalone, *Haliotis rubra* and greenlip abalone, *H. laevigata*) inhabiting coastal South Australia, using multiple species distribution models (SDM) and for decadal time slices through to 2100. Projections are based on two contrasting global greenhouse gas emissions scenarios. The SDMs identified August (winter) Sea Surface Temperature (SST) as the best descriptor of abundance and forecast that warming of winter temperatures under both scenarios may be beneficial to both species by allowing increased abundance and expansion into previously uninhabited coasts. This range expansion is unlikely to be realised, however, as projected warming of March SST is projected to exceed temperatures which cause up to 10-fold increases in juvenile mortality. By linking fine-resolution forecasts of sea surface temperature under different climate change scenarios to SDMs and physiological experiments, we provide a practical first approximation of the potential impact of climate-induced change on two species of marine invertebrates in the same fishery.

## Introduction

The distributions of many marine species are strongly related to environmental conditions, making them susceptible to both medium- and long-term changes in abiotic conditions (e.g. medium-term changes to El Niño cycles: [1]; and long-term climate change: [2],[3]). Commercially exploited populations may be particularly susceptible to changes in environmental conditions, as many of the world's fisheries stocks are over-exploited, with benthic habitats also being damaged by fishing operations [4], and consequently the capacity of their populations to withstand multiple and interacting environmental changes is eroded [5].

Current projections suggest that multi-decadal increases in ocean temperatures are likely to cause commercially harvested species to become increasingly vulnerable to overfishing at lower latitudes, where species are closer to thermal-tolerance limits and have been forecast to undergo population declines [6]. Such predictions are supported by observations of different populations of the same fish species at high and low latitudes alternately experiencing either population increase or decrease with historical warming (e.g. Norwegian cod; [7],[8]). Current modelling and empirical techniques can be used to predict the geographical range of marine species at coarse scales [9], [10] and in rare instances have been used to detect present-day spatial abundance patterns at finer resolutions (e.g. [11]). Yet, future projections of changes in range and abundance have been limited by uncertainties arising from choice of species distribution model (SDM) and global climate model (GCM), a lack of consideration of model-selection uncertainty, and a failure to integrate relevant biological detail (e.g., species interactions, connectivity and dispersal). All of these factors influence projections of species' range movement and extinction risk [12], [13]. Therefore, efforts to address these model-related uncertainties should lead to improvements in the capacity to predict the abundance of commercially harvested species under changing environmental conditions, and may enhance the capability of management to ensure harvest can be maintained into the future.

Research on the causes of and changes in the distribution of marine species, to date, has been limited by a paucity of high-resolution and broad-scale environmental data in the marine realm [14], [15]. In cases where climate-change impacts have been assessed, they have mostly focused on bioclimatic envelop or single species distribution model (SDM) approaches [14], sometimes integrating demographic information [6]. While these approaches can predict present-day distributions reasonably well, projections of future distributions can vary widely among SDMs [16], [17], [18]. Recent advances in multi-model ensemble techniques may provide more robust and realistically bounded estimates of future distributions [19], but have only recently been applied in marine systems (e.g. [11]).

Further complicating such predictions are uncertainties in the climate projections themselves. The degree of confidence that we can place on future climate change projections depends on GCM performance and uncertainties that need to be assessed rigorously, via climate model evaluation [20]. GCMs are helpful in providing physically realistic representations of global-climate dynamics [21], yet they tend to provide less reliable descriptions of local and regional climates [22], partly because processes that occur at scales smaller than the GCM resolution (such as cloud and topographic influences) cannot be modelled explicitly and must be parameterised [23].

Here, we use two species of commercially harvested abalone with overlapping ranges, blacklip (*Haliotis rubra*) and greenlip (*H. laevigata*), to demonstrate a novel correlative and experimental approach for predicting the future distribution and abundance of marine invertebrates. While present-day management of the fishery is appropriate [24], [25], forecast changes in key environmental requirements may mean that fine-scale estimates of population densities under future conditions will be required to successfully manage the long-term persistence of abalone stocks. At the moment climate change is not being considered in the fishery management plans. We hypothesise that green and blacklip abalone will respond differently to future ocean warming, because the two species have different biological responses to temperature, with blacklip exhibiting a lower optimal temperature than greenlip (17.0°C *vs.* 18.3°C, respectively; [26]), but above a certain thermal tolerance this difference is likely to disappear. While recently developed SDMs have identified the most likely environmental factors which drive population densities, including temperature [11], there are currently no forecasts of how these populations will change under future climates.

Thus, the objectives of this study were to: (1) project changes in the range and abundance of *H. rubra* and *H. laevigata* in southern Australia at decadal time slices under different climate-change scenarios; (2) develop forecasts of sea surface temperature which better account for inter-model variation in GCM projections, downscaled to biologically relevant resolutions; and (3) determine whether demographic processes need to be incorporated in models by experimentally assessing juvenile mortality at projected temperatures.

## Materials and Methods

### Species distribution models

Blacklip (*Haliotis rubra*) and greenlip (*H. laevigata*) abalone occur on the southern coast of Australia and have overlapping distributions (greenlip abalone occupying approximately the central 2/3 of the distribution of blacklip [27]). For the specific purpose of this study, a species' range refers to its area of occupancy within the study area, i.e. corresponding to the spatial extent of the fishery in southern Australia. Distribution and abundance data for both species within their ranges were collated from multiple fisheries monitoring and regional biodiversity surveys between 1980 and 2009. All surveys were done using SCUBA on rocky substrate between 5–30 m depth across South Australia (approximately 130–142°E). In previous work, we modelled the present-day distribution and spatial abundance of both species individually using SDMs [11]. To summarise (as background to the current forecast-based paper), a multi-model ensemble averaging technique was used to weight SDM projections. Generalized linear models (GLM) and boosted regression trees (BRT) were used to generate the model-averaged forecasts of abundance, because both techniques demonstrated good skill in forecasting present-day occurrence and abundance patterns for *H. rubra* and *H. laevigata* (see [11] for more comprehensive detail on modelling abundance with these SDMs). We constrained model development and training to the region with the most comprehensive abundance survey data available (South Australia). Out-of-sample validation included an assessment of spatial transferability of model predictions; SDMs were validated using independent data from similar surveys done across several hundred km of coast to the east of the study area (approximately 142–147°E).

For both abalone species, the best primary predictors of abundance were mean August (winter) sea surface temperature (SST) and its standard deviation (a linear correlative relationship for *H. rubra*, quadratic for *H. laevigata*), harvest intensity, water depth and distance from the nearest boat launch point. While sea bottom temperature may be slightly cooler than SST (<2°C at depths less than 30 m [mean difference = 0.27°C]) in our study region, bottom temperature was strongly correlated with SST (r = 0.825) and was not a better predictor of abalone abundance than SST [11]. Therefore, we chose to use SST as our primary temperature predictor in the model. To determine whether anticipated change in future SST can be expected to influence the range dynamics of either abalone species, we developed downscaled-decade forecasts of August SST (2010–2100), according to different greenhouse gas emissions scenarios. All other predictors were fixed to the values used for model fitting [11] for a particular location.

### Sea Surface Temperature projections

The SST data were extracted from satellite images focused on southern Australia at a 4.6-km resolution (AVHRR Pathfinder product version 5.0), for separate day and night passes. While sea surface temperatures are often higher than those near the sea bottom, AVHRR satellite derived SST data correlate well with bottom temperatures [28]. Mean monthly day and night SST data from 1985 to 2004 were used to calculate a 20-year monthly day/night average for August and March SST [11]; a period that closely resembles the baseline period used to validate GCMs (1980–2000). Thin-plate–spline surface-smoothing techniques were then used to downscale the coarse-resolution data to a 0.01° latitude/longitude grid-cell resolution [11]. The degree of smoothness of the fitted function was determined by minimizing a measure of predictive error of the fitted surface given by the generalised cross validation [29]. Moreover, out-of-source sampling on a subset of the Pathfinder data, specifically retained for validation, was used to evaluate model fit (<±0.5°C) [11]. MAGICC/SCENGEN 5.3 (http://www.cgd.ucar.edu/cas/wigley/magicc), a coupled gas cycle/aerosol/climate model used in the IPCC Fourth Assessment Report [30], was used to generate future changes in August and March SST at the turn of each decade (2010–2100) using an ensemble of five GCMs, chosen according to their superior skill in globally forecasting March, August and annual SST and their consistency with other GCMs [20], [31]. The skill of the full suite of GCMs used for the Fourth Assessment Report (AR4) of the Intergovernmental Panel on Climate Change (IPCC) can be assessed directly in MAGICC/SCENGEN according to their ability to simulate observed conditions using different variables, and different statistical-validation metrics, over any user-specified region.

The comparison metrics that we used for validation of GCM outputs were: (i) model bias (i.e., the difference between model and observed spatial means averaged over a user-specified area); (ii) pattern correlation; and (iii) standard and centred root-mean-square errors. Rather than using actual values of these various statistics, we placed them on a level playing field by using only model ranks for each statistic. Our key overall comparison metric was the cumulative rank [31]. MAGGICC/SCENGEN also allows for an outlier analysis to be computed which compares future projections based on individual models with the average projection of all other models. Although, GCM data from the Coupled Model Intercomparison Project 3 (CMIP3) archived GCM database (www-pcmdi.llnl.gov/) could be used to generate ensemble averaged forecasts directly, a key features that makes working within the MAGICC/SCENEGEN framework superior is the way in which MAGICC/SCENEGEN standardises different GCMs to align to user-specified selected climate sensitivities, meaning inter-model differences in future climate forecasts can be studied without being obfuscated by differences in climate sensitivity [31].

The five best-ranked models were CCSM3, MIROC 3.2 (hires), ECHAM5/MPI-OM, MRI-CGCM2.3.2 and GFDC-CM2.1 (model terminology follows that used in the CMIP3 model data base). These five models were used to generate multi-model averaged climate forecasts – change in average daily SST (°C) in August and March (2.5×2.5° latitude/longitude grid cell resolution). With less than five models the results are more sensitive to the number and choice of models, while for more than five models, the additional information has a relatively small effect on the average forecast [32]. Climate forecasts were generated according to two emission scenarios: a high-CO_2_-concentration stabilising Reference scenario (WRE750) and a more conservative Policy emissions scenario, assuming substantive intervention (LEV1) [33], [34].

These climate anomalies were downscaled to an ecologically relevant spatial scale (0.01×0.01° longitude/latitude), using the “change factor” method, where the low-resolution change from a GCM is added directly to a high-resolution baseline observed climatology [35]. Bi-linear interpolation of the GCM data (2.5×2.5°) to a resolution of 0.5×0.5° longitude/latitude was used to reduce discontinuities in the perturbed climate at the GCM grid-box boundaries [20]. While there is a range of alternative approaches, the simple “change factor” approach that we advocate is easily implemented in such a way that uncertainties arising through the generation of the baseline layer and overlay process can be easily documented.

### Abalone range projections

To better understand the potential impact of forecast changes in August SST on the range and abundance of abalone, we used our already established ensemble SDM modelling approach [11] to project the spatial abundance of *H. rubra* and *H. laevigata* at the turn of each decade (2010–2100). We present forecast change in abundance (number of individuals per 100 m^2^), and mean percent change in abundance above a minimum threshold of 20 individuals/100 m^2^, for both *H. rubra* and *H. laevigata* for 2100. The 20 individuals/100 m^2^ threshold was chosen because this is the minimum density needed to maintain the rates of recruitment required to sustain catches [36], [37]. We also map changes in potential fishing grounds for *H. rubra* and *H. laevigata* in 2100 according to both emission scenarios.

### Juvenile mortality - experimental methods

To test whether realised range expansion was likely to be reduced by elevated March SST, the effects of elevated temperature on juvenile *H. laevigata* mortality was experimentally tested. Mortality of juvenile abalone was recorded in a laboratory experiment spanning one month to match the monthly average temperature used in model projections. Two temperatures were used in the experiment, 17°C and 20°C, as they represent the lower and upper March temperature categories across the current distribution of *H. laevigata*; densities decline above and below these thresholds (see results below). Experiments were done on *H. laevigata* because juveniles were readily available (KIAB aquaculture, Kangaroo Island, South Australia) and data were available for *H. rubra* [38].

Experiments were conducted in 44 L aquaria with water constantly recirculating from a 200 L reservoir beneath each tank. There were 4 replicate aquaria per temperature and 8 replicate individual abalone per tank. A pump moved water from the reservoir at a constant flow rate of 200 L hr^−1^ to the tank. To maintain good water quality (i.e. nutrients and salinity), 50% of the water in each set-up was replaced weekly with fresh seawater. Light was provided in a 12∶12 light dark cycle by pairs of fluorescent lights above each tank. Each light had one “grow light” which incorporated the UV spectrum (Sylvania® Gro-lux) and one “daylight” (Luxling® Daylight deluxe). Each tank contained rocks covered in coralline crusts and turf-forming algae to represent a natural environment. Abalone were fed a 1–3 mm formulated feed (EP Aquafeeds, Lonsdale, South Australia) every second day (17:00 hrs). Any unconsumed feed was removed from tanks the following morning (09:00 hrs). All tanks were aerated, with a constant flow of 10 L min^−1^. Temperature levels remained constant throughout the treatment period. Elevated temperature (20°C±0.5°C) was controlled using Aqua One aquarium heaters. Ambient temperature (17°C±0.5°C) was controlled by recirculating water through chilling units (TECO-Ravenna). Experiments were done in a constant temperature laboratory to ensure no external inputs created temperature fluctuations and temperature was measured daily to ensure treatment levels were achieved.

## Results

Under the Reference scenario (WRE 750; higher-CO_2_-emission stabilisation), August (winter) SST is predicted to consistently increase over the next century to be ∼1.1°C higher by 2100 (Fig. S1a). Under the Policy emissions scenario (LEV1) which assumes strong greenhouse-gas mitigation, however, average August SST in the study region is projected to increase by ∼0.46°C by 2080 and then reduce to ∼0.44°C above 1994 temperatures by 2100 (Fig. S1a). Warming will not be spatially consistent across the study area, however, with temperatures being between 0.87–1.77°C and 0.37–0.85°C higher in 2100 than 1994 for the Reference and Policy scenarios, respectively.

**Figure 1:**
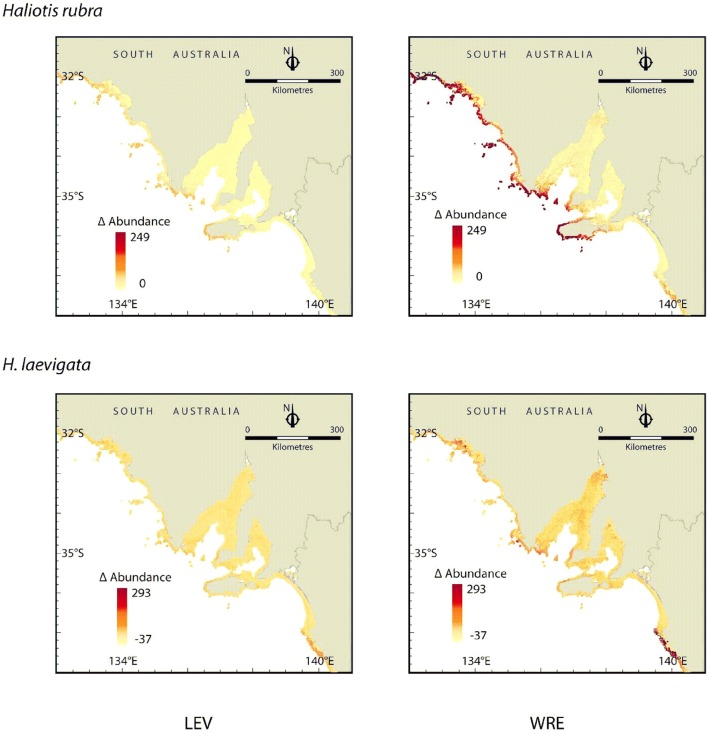
Forecast change in the abundance (number of individuals per 100 m^2^) of *Haliotis rubra* (blacklip abalone) and *H. laevigata* (greenlip abalone) by 2100 based on projections of August SST according to two climate change emissions scenarios: a high CO_2_ concentration stabilising scenario (WRE750) and a heavy mitigation Policy option (LEV1).

SDM future projections indicate that under the Reference scenario, both species are predicted to increase in abundance in response to increased August SST, albeit blacklip to a greater degree. Under this scenario, blacklip abundance is predicted to increase by up to 2.5 individuals m^−2^ across approximately the western two-thirds of their distribution in South Australia, while areas of substantial increases in abundance of greenlip abalone (approximately 2.5–3 indiv. m^−2^) were more restricted to the south-eastern part of their range (Fig. 1). Elevated August SST can be expected to increase blacklip harvestable biomass by >40% by 2100 under this Reference scenario (Fig. S1b). In contrast, the more geographically restricted increase in forecasted abundance for greenlip abalone resulted in a relatively small projected increase in harvestable biomass by 2100, approximately 15% above present day levels (Fig. 1). Under the lower emission Policy scenario, SDM projections indicate that neither species of abalone will show substantial changes in abundance by 2100 (Fig. 1). This small predicted increase from current-day abundance would translate into little change in the harvestable biomass by 2100, with only marginal increases above a critical sustainable harvesting density (0.2 reproductive adults m^−2^) of approximately 20% and <10% for blacklip and greenlip, respectively (Fig. S1b).

**Figure 2:**
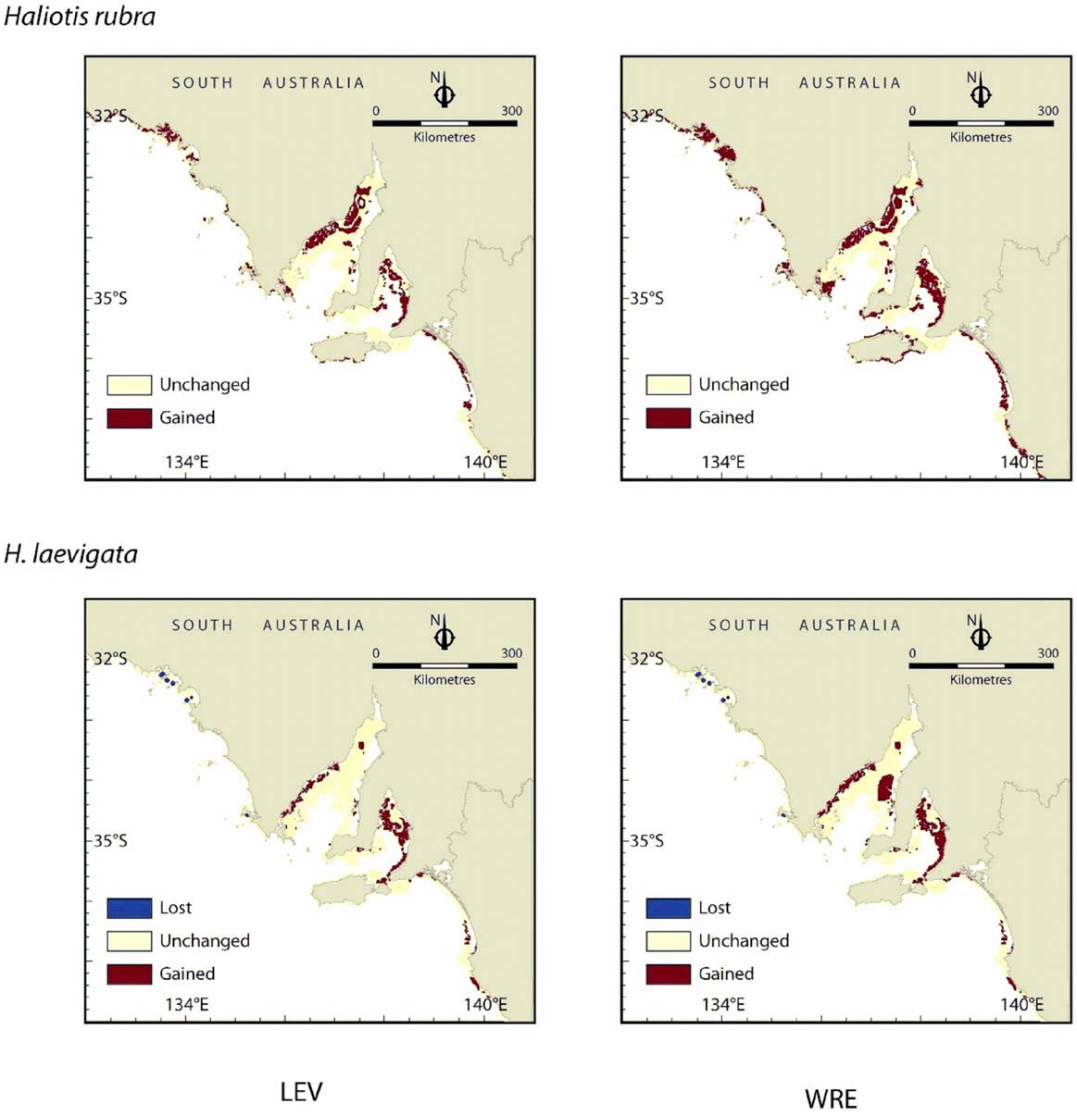
Changes in potential fishing grounds for *Haliotis rubra* (blacklip abalone) and *H. laevigata* (greenlip abalone) in 2100 based on projections of August SST according to two climate change scenarios: a high CO_2_ concentration stabilising scenario (WRE750) and an alternative scenario that assumes strong mitigation (LEV1). Potential fishing grounds are defined based on a minimum abundance of 20 individuals/100 m^2^.

Overall, the SDMs predict expansion of potential fishing grounds for both species by 2100 based on predicted increases to August SST (Fig. 2). Blacklip fishing grounds would be gained across much of their range in South Australia, with greater expansion under the Reference scenario (Fig. 2). Expansion would be less for greenlip abalone and there would also be a loss of a small area of fishing grounds in the western part of South Australia (Fig. 2).

**Figure 3:**
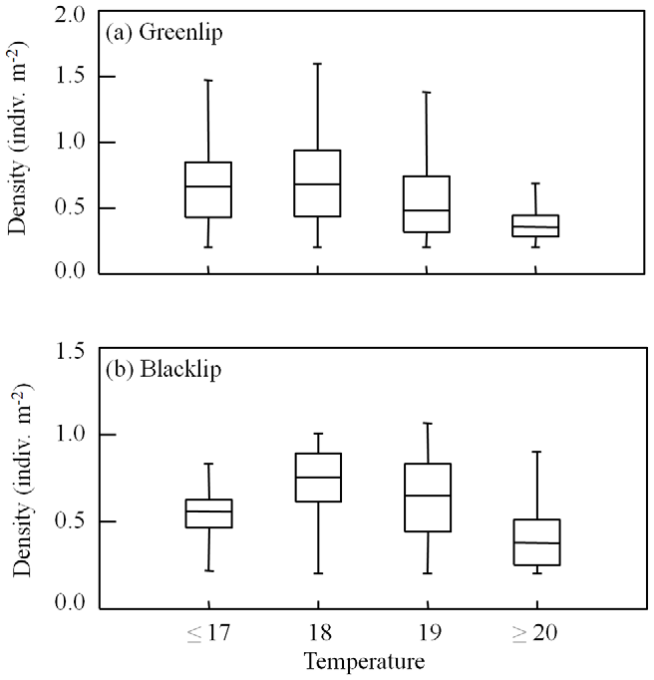
Box plots of the density of (a) greenlip and (b) blacklip abalone within their current distribution in South Australia, categorised according to average March Sea Surface Temperatures. Note the decrease in density of individuals for both species at 20°C and above.

The present-day density of both species of abalone varies with summer (as well as winter) maximum monthly temperature across their current distribution. Density is greater in locations (1 km×1 km grid cells) where average March SST exceeds 17°C, but then declines substantially in locations where March temperatures are at or above 20°C (Fig. 3 a & b). In the laboratory experiment, mortality of juvenile greenlip abalone was over 10 times greater at 20°C (mean ± SE; 58.12%±8.6) than at 17°C (3.1%±3.1) (one-way ANOVA: F_1,6_ = 35.96, *p* = 0.001). This result is supported by the mean March SST at the distribution edges of both species for the same period used to train the SDMs. Mean temperatures at the north-eastern and north-western distribution limits of blacklip were 23.6°C and 19.9°C, respectively, with greenlip being 19.2°C and 19.0°C, respectively.

**Figure 4:**
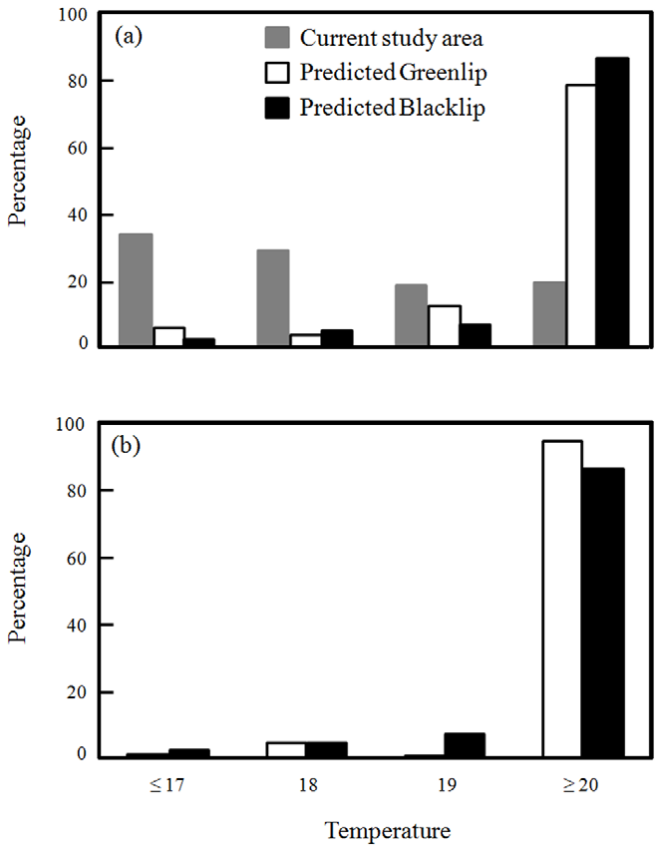
The percentage of South Australian waters at different mean-March temperatures. (a). Grey bars show current-day temperatures across the entire area, while the white and black bars show the percentage of the current distributions of greenlip (white bars) and blacklip abalone (black bars),at predicted temperatures for 2100 under the high-CO_2_ Reference scenario and (b) the percentage of predicted abalone distributions (based on SDMs using August SST) which would be at different March temperatures in 2100. The current day temperatures (grey bars) are the same for both (a) and (b). Also note for both (a) and (b) the increase percentage of the area at or above 20°C, meaning that these predicted distributions may not be realised.

Within a large proportion of the study area (∼33%), average March temperatures are currently at or below 17°C with less than 20% of the area having temperatures at or above 20°C (Fig. 4a). Under the Reference scenario, the majority of the current distribution of both species is predicted to be at or above 20°C (∼78% and ∼86% for green- and blacklip, respectively), with only a small percentage of their distribution at or below 17°C (∼5% and ∼2%, respectively; Fig. 4a). Of the locations (1 km×1 km grid cells) which the SDMs predicted would show expansion of abalone populations, ∼94% and 86% are predicted to have average March SST of at or above 20°C by 2100 for green- and blacklip, respectively (Fig. 4b).

## Discussion

We predict, based on correlative SDM projections, that both blacklip and greenlip abalone could increase in abundance and expand into new locations in response to warming winter temperatures under both high and low high CO_2_ concentration stabilisation scenarios. However, projections of temperatures in warmer months (March), viewed in conjunction with experimental data, suggests that much of the predicted area of population expansion would be too hot, thus limiting realised range expansion through elevated juvenile mortality. Thus, to accurately predict the potential future distribution of species, we need to understand physiological responses of species across all of their life stages [39] and develop modeling techniques that can make the best use of this information.

By using SDMs coupled to downscaled multi-model ensemble averaged climate projections we were also able to identify that there will be species-specific responses to warming, with blacklip populations predicted to expand across their South Australia but greenlip ones to a lesser degree, only expanding in the eastern part of the South Australian fishery. These predictions of range expansion are not isolated to abalone. Increasing winter temperatures in the North Sea over the past three decades correlated well with the northward movement of a range of taxonomically diverse species, including deep-sea fish [9], intertidal [3], [40] and subtidal invertebrates [41] and their algal habitats [42]. Our models suggest an increase in winter temperatures could allow both blacklip and greenlip abalone to increase abundance within their distribution in South Australia, as well as expand into unoccupied habitat. However, the SDMs did not incorporate the concomitant increase in March SST (because March SST was not a strong predictor of present-day abundance; [11]), yet projections of these warmer temperatures suggest that the majority of the predicted area of population expansion would be too hot, thus limiting realised range expansion (Figure 4).

Reproductive output and survival of recruits are key factors that determine the range of a species. In our case study, August (winter) temperature had the largest (positive) influence on the distribution of both species of abalone [11], which is expected to increase in response to a low-, as well as high-CO_2_ concentration stabilizing scenario, promoting range expansion, albeit to a lesser degree for the more conservative emission Policy scenario. While recognising that the techniques we employed were correlative, we can speculate regarding possible elements of causation underpinning the model projections. The most likely mechanisms for the SDMs predicting population expansion under future warming would be increases in both reproductive potential of the adults and survival of recruits in winter. Both species of abalone used in this study show a linear relationship between the rate of gonad development and temperature between 12–18°C [43], leading to a greater reproductive output and development of larvae [44], promoting faster settlement and increased chance of survival. Concurrently, higher winter and spring temperatures improve survival in juveniles of many species of mollusc (e.g. scallops, [41]; abalone, [45], [46]).

It is unlikely, however, that the trend in global warming will be consistent among the seasons [47], and the same warming that may increase the reproductive output and recruitment of abalone in winter may also drive them above thermal tolerances in summer. This context dependence has been recognised in temperate oceans of the northern hemisphere; warming of the ocean off the Norwegian coast in spring increases growth and survival of juvenile cod, while warmer temperatures in summer increase metabolic costs and reduces growth [8]. Further, this relationship is likely to vary with latitude, as most species tend to be more susceptible to increasing temperatures in lower latitudes because they are closer to their physiological limits (e.g. [7].

Under current seasonal temperature ranges, the summer maximum temperature in the study region is below the thermal maxima of both species [26]. However, projections of March (warmer) sea surface temperatures suggest that the majority of the areas of predicted population expansion for both species would be above 20°C (Figure 4b). Water temperatures >20°C in laboratory experiments (greenlip, this study; blacklip, [38]) caused a 10-fold increase in juvenile mortality. While temperature close to the sea bed would be lower than that of the sea surface, potentially reducing mortality below that seen in experiments at 20°C, this is offset by mortality tending to be higher in the natural environment than experimental studies [48]. Additionally, mean March SST at the current distribution limits of greenlip abalone (19.2°C and 19.0°C) support the idea that increasing March water temperatures may counter the biological benefits of increasing winter temperatures. This interpretation is supported by the current densities of both species, which are low in areas with average March SST above 20°C (Figure 3).

Since abundance-type model projections are often used to inform fisheries managers of sustainable commercial catches, it is important that projection uncertainties are explored and, where possible, minimized. Our SDMs predict both an expansion into unoccupied habitats and an increase in the density of individuals within current fishing grounds, which could be interpreted as increased harvest in these locations or greater population densities at current harvest levels. In other abalone species years of higher temperature anomalies (increases) have meant greater catches because of greater recruitment [49]. However, because a large proportion of the populations of both species in South Australia are likely to experience March SST above 20°C in the future (Figure 4), we expect that any positive effect of warmer winters on abalone abundance would be nullified by a concomitant increase in March temperatures causing greater juvenile mortality. As such, the SDM projections may represent an overestimation of the potential expansion of abalone into new fishing grounds, with any realised range expansion being substantially less.

**Figure 5:**
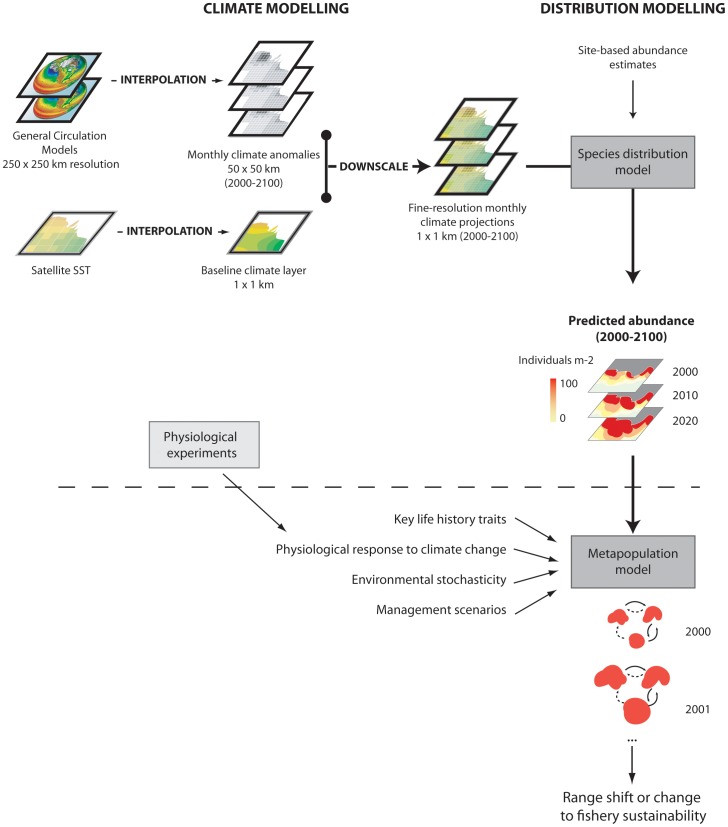
Schematic diagram of a hybrid-modelling approach to identify potential climate-driven changes in the distribution and abundance of commercially harvested species, and to test different fisheries management scenarios. The modelling steps that have been completed are located above the dotted line. The next step is to couple this approach with spatially explicit stochastic-demographic models, to capture some of the complexities and uncertainties underlying biological mechanisms driving species distribution and abundance patterns in response to forecasts of future climate change and harvest pressure.

By downscaling multi-model-averaged climate forecasts to a fine resolution and generating annual projections, we improved forecasts of the influence of climate change on green and blacklip abalone range and abundance (i.e., through more robust forecasts of SST at local spatial- and short temporal-scales [50]). Recent laboratory research (greenlip, this study; blacklip, [38]), however, shows the importance of explicitly incorporating demographic processes (e.g. juvenile mortality and recruitment) into climate impact assessments. We suggest that predictions of the effect of future climate on abalone range dynamics would be strengthened by using a simulation framework that couples SDM forecasts to structured spatial population models (e.g. Figure 5). A caveat is that these sorts of models are data intensive, requiring a strong understanding of the population dynamics of the focal species (typically linked to long-term monitoring programmes, including measurements of vital rates), as well as high-resolution distributional data [51] which needs to be ground-truthed to validate the accuracy of underlying distribution models. In this respect, commercially harvested species provide a good test case because the intensive study of their biology over many years generally makes this data available.

In this case study, the environmental conditions which best predicted the distribution and abundance of abalone in the SDMs under current conditions (August SST) may not accurately predict future populations if concomitant warming of summer temperatures reduces the realised distribution. Thus, robust forecasts of fisheries species need to incorporate metapopulation processes, such as spatially and temporally variant recruitment rates [52]. While other marine studies have found similar results on large scales [e.g. 30′×30′ grids; 6], we provide an important advance towards predictions on a scale that is relevant to management of fisheries (i.e. 1 km×1 km grids).

## Supporting Information

Figure S1 (a) Forecasts of mean August sea surface temperature across the study area based on two climate change scenarios: a high CO_2_ concentration stabilising scenario (WRE750) and a more conservative scenario, assuming heavy CO_2_ mitigation (LEV1). The mean SST in the study area for 1994 (baseline for the forecasts) is also shown. Error bars show the standard deviation within the study area. Note that the WRE750 data points have been offset for clarity. (b) Forecast mean precent change in the abundance of *Haliotis rubra* (blacklip abalone) and *H. laevigata* (greenlip abalone) above a minimum threshold of 20 individuals/100 m^2^, according to a high CO_2_ concentration stabilization Reference scenario (WRE 750) and a heavy mitigation Policy option (LEV1).
(DOCX)
