# Supplementary material for: Predicting the Distribution of Commercially Important Invertebrate Stocks under Future Climate
Source: PLoS One. 2012 Dec 12;7(12):e46554. doi: 10.1371/journal.pone.0046554 (PMC3520996; doi:10.1371/journal.pone.0046554)
Supplement: Figure S1 — (a) Forecasts of mean August sea surface temperature across the study area based on two climate change scenarios: a high CO2 concentration stabilising scenario (WRE750) and a more conservative scenario, assuming heavy CO2 mitigation (LEV1). The mean SST in the study area for 1994 (baseline for the forecasts) is also shown. Error bars show the standard deviation within the study area. Note that the WRE750 data points have been offset for clarity. (b) Forecast mean precent change in the abundance of Haliotis rubra (blacklip abalone) and H. laevigata (greenlip abalone) above a minimum threshold of 20 individuals/100 m2, according to a high CO2 concentration stabilization Reference scenario (WRE 750) and a heavy mitigation Policy option (LEV1). (DOCX) [file pone.0046554.s001.docx]

**Supporting information**

Figure S1. (a) Forecasts of mean August sea surface temperature across the study area based on two climate change scenarios: a high CO_2_ concentration stabilising scenario (WRE750) and a more conservative scenario, assuming heavy CO_2_ mitigation (LEV1). The mean SST in the study area for 1994 (baseline for the forecasts) is also shown. Error bars show the standard deviation within the study area. Note that the WRE750 data points have been offset for clarity. (b) Forecast mean precent change in the abundance of *Haliotis rubra* (blacklip abalone) and *H. laevigata* (greenlip abalone) above a minimum threshold of 20 individuals/100m^2^, according to a high CO_2_ concentration stabilization reference scenario (WRE 750) and a heavy mitigation policy option (LEV1).

Fig. S1
